# Baltimore College of Dental Surgery

**Published:** 1849-07

**Authors:** 


*Baltimore College of Dental Surgery.*—In compliance with the request of most of the gentlemen composing the Examining Committee, the Faculty of this institution have determined to hold the commencements, hereafter, the last, instead of the first, of March; consequently, the course will commence nearly a month later for the future, than formerly. The Mechanical, Infirmary and Dissecting rooms will be opened, as may be seen by the advertisement at the back of the Journal, the last Monday in October, and the Lectures will commence the last Monday in November.—*Balt. Ed.*

